# Conceptualizing nurses’ innovative behavior: a qualitative content analysis of process and dimensions

**DOI:** 10.1186/s12913-026-15320-5

**Published:** 2026-08-14

**Authors:** Elham Shahidi Delshad, Mohsen Soleimani, Armin Zareiyan, Ali Asghar Ghods

**Affiliations:** 1 Department of Anesthesia, School of Allied Medical Sciences, Tehran University of Medical Sciences, Tehran, Iran; 2 Nursing Care Research Center, Semnan University of Medical Sciences, Semnan, Iran; 3 Research Center for Cancer Screening and Epidemiology and Health in Disaster and Emergencies Department, Nursing Faculty, Aja University of Medical Sciences, Tehran, Iran; 4 Critical Care Nursing Department, School of Nursing and Midwifery, Semnan University of Medical Sciences, Semnan, Iran

**Keywords:** Innovative behavior, Nursing innovation, Qualitative research, Content analysis, Nursing practice, Innovation process, Nursing leadership

## Abstract

**Background:**

Increasing healthcare demands and rapid technological advancements have intensified pressures on hospital organizations, highlighting the critical role of nurses’ innovative behavior in ensuring adaptive and patient-centered care. However, existing research is predominantly quantitative and offers limited insight into the underlying dimensions and processes through which innovation is enacted in clinical practice. This study aimed to qualitatively conceptualize nurses’ innovative behavior from the perspective of clinical nurses.

**Methods:**

A qualitative descriptive study was conducted using conventional content analysis. Data were gathered through semi-structured, face-to-face interviews with 14 clinical nurses recruited via purposive sampling from diverse hospital settings. An 8-step analytical framework (Zhang & Wildemuth) was employed to systematically code and categorize the data, ensuring methodological rigor and transparency throughout the analytical process.

**Results:**

The analysis revealed five interrelated dimensions of nurses’ innovative behavior: nurses’ competencies, generation of care ideas, feasibility of care ideas, clinical implementation, and promotion of innovation.

**Conclusion:**

Nurses’ innovative behavior is primarily driven by professional necessity, the everyday clinical challenges at the bedside, and often manifests as informal, invisible daily practice. By rendering this invisible work visible, the findings provide a grounded conceptual framework for refining measurement instruments and fostering innovation cultures, particularly in resource-constrained healthcare settings.

**Supplementary Information:**

The online version contains supplementary material available at https://doi.org/10.1186/s12913-026-15320-5.

## Introduction

Healthcare systems worldwide are experiencing unprecedented pressure due to the rapid growth of population health needs, increasing patient complexity, and accelerated advancements in health technologies [1]. Hospital organizations are required to respond simultaneously to rising demands for care, stringent regulatory requirements, and persistent financial constraints, while maintaining quality, efficiency, and value-based outcomes [2, 3]. Within this increasingly complex environment, organizational sustainability and performance are no longer achievable through routine practices alone. Instead, the capacity of healthcare organizations to adapt, evolve, and innovate has become a strategic imperative. Innovation generally refers to the process of generating, introducing, and applying new or significantly improved ideas, practices, services, or processes that create meaningful value within a specific context [4]. In healthcare settings, innovation extends beyond technological advancement and includes novel approaches to care delivery, communication, workflow organization, and problem-solving aimed at improving patient and organizational outcomes [5]. In this context, employees’ innovative behavior has emerged as a critical driver for addressing patient needs, responding to environmental changes, and facilitating the generation and adoption of novel ideas in healthcare delivery [6, 7]. Innovative behavior is therefore not merely a reactive response to systemic challenges, but a proactive mechanism for shaping the future of healthcare systems and improving population well-being [8, 9]. The growing integration of artificial intelligence and machine learning into clinical practice, largely enabled by innovative healthcare professionals, illustrates how individual-level innovation can translate into system-level improvements, such as enhanced diagnostic accuracy and personalized treatment pathways [10, 11].

As the largest group of frontline healthcare professionals, nurses occupy a unique and influential position in translating innovation into everyday clinical practice. In the present study, the term clinical nurses refer to registered nurses holding bachelor’s or master’s degrees in nursing who are directly engaged in patient care within hospital settings. Nurses’ innovative behavior plays a pivotal role in advancing health promotion, mitigating disease-related risk factors, preventing illness, promoting healthy lifestyles, and continuously refining care processes and clinical protocols [12, 13]. Conceptually, nurses’ innovative behavior encompasses their proactive, adaptive, and intentional engagement in generating, refining, implementing, and disseminating novel ideas, practices, or approaches aimed at improving patient care, health outcomes, and the effectiveness of healthcare delivery systems [14, 15]. Empirical evidence demonstrates that nurses contribute to innovation across multiple domains of care. In health promotion, nurses have developed and implemented creative interventions that extend beyond traditional clinical settings [16], while in disease prevention, their innovative practices increasingly incorporate community-based and population-focused strategies [17]. Moreover, nurses’ ongoing engagement with evidence-based practice and emerging technologies supports the development of patient-centered and responsive models of care [8, 18, 19]. Collectively, these innovative efforts enable healthcare systems not only to respond to current demands but also to anticipate and adapt to future challenges [2, 20].

Despite the growing recognition of the importance of nurses’ innovative behavior, the existing body of literature is characterized by a strong predominance of quantitative research designs. Most studies have focused on examining the relationships between nurses’ innovative behavior and related constructs such as leadership styles, organizational culture, psychological empowerment, and job characteristics [21, 22]. While these studies provide valuable insights into correlational patterns and predictive factors, they offer limited understanding of how innovative behavior is conceptually structured, enacted, and experienced by nurses within real-world clinical contexts. As a result, nurses’ innovative behavior remains insufficiently explored in terms of how it is experienced, interpreted, and enacted within real-world clinical contexts.

This gap is particularly consequential given the increasing use of standardized measurement instruments to assess innovative behavior in nursing populations. Without a robust qualitative understanding of how nurses themselves conceptualize and operationalize innovative behavior, existing instruments risk oversimplifying the construct or failing to capture dimensions that are salient in clinical practice. Qualitative inquiry is therefore essential for uncovering the underlying dimensions, processes, and meanings of nurses’ innovative behavior as embedded within specific healthcare contexts. By systematically exploring nurses lived experiences, perspectives, and narratives, qualitative research can provide the conceptual clarity required for theory refinement and the development of context-sensitive measurement tools [23].

Accordingly, the present study aimed to explore and qualitatively conceptualize nurses’ innovative behavior from the perspective of clinical nurses. By adopting a conventional content analysis approach, this study sought to generate a practice-grounded understanding of how innovative behavior is experienced and enacted in clinical settings.

## Methods

### Objective and research question

The objective of this study was to qualitatively conceptualize nurses’ innovative behavior by exploring the lived experiences and perspectives of clinical nurses, with the aim of identifying its key dimensions and underlying processes as enacted in clinical practice.

Research Question: What dimensions and subcategories constitute nurses’ innovative behavior in clinical practice?

### Research design

This study employed a qualitative descriptive design guided by the Conventional Content Analysis approach, as outlined by Zhang and Wildemuth (2016, Chap. 18) [24]. This methodological framework was selected to allow the dimensions and processes of nurses’ innovative behavior to emerge inductively from the participants’ lived experiences, rather than being constrained by existing quantitative frameworks. All interviews were conducted in a face-to-face format to facilitate the establishment of rapport and to capture the non-verbal cues essential for a deep conceptualization of the phenomenon. We avoided online or telephone modalities to ensure the participants felt sufficiently secure and engaged to share their personal narratives in their natural clinical and professional environments.

### Participants

The research utilized purposive sampling and adhered to specific inclusion criteria: (1) recruitment of registered clinical nurses working in hospital settings and directly involved in patient care, (2) requirement of a minimum of one year of professional experience, (3) possession of a Bachelor of Science (BSN) degree or higher in nursing, and (4) demonstration of willingness to participate in the study.

Participants were recruited through a combination of direct invitations by the research team during visits to hospital wards and via professional nursing networks. To maintain the autonomy of participants and mitigate potential organizational bias, recruitment was performed independently by the research team, rather than through direct referrals by hospital managers or administrators. This independence was emphasized during the recruitment process to assure participants of the confidentiality and voluntary nature of their involvement.

### Data collection

This qualitative study was conducted between November 2022 and August 2023. Semi-structured, face-to-face interviews were employed to gather in-depth data regarding nurses’ innovative behaviors. Interviews were conducted in the Persian language to ensure participants could express their experiences authentically.

### Setting and fieldwork

To capture maximum variation in experiences and organizational contexts, participants were recruited from both large academic teaching hospitals and private healthcare institutions in Tehran. These settings differed in terms of organizational structure, patient populations, clinical workload, and care delivery priorities, thereby providing diverse perspectives on nurses’ innovative behavior across distinct healthcare environments.

### Interviewing strategy

A semi-structured interview guide [24] was developed by the research team. The English version of the interview guide is provided as Supplementary File 1. Sessions began with warm-up questions designed to establish rapport and invite the sharing of personal narratives. As the study progressed, questions were refined to become more specific based on emerging insights. We employed probing techniques, such as “Could you explain that further?” or “Could you provide an example?“, to facilitate depth. The interview duration ranged from 50 to 95 min (mean: 72 min).

### Transcription and data preparation

Transcriptions were generated verbatim in Persian immediately following each interview to preserve the fidelity of the original discourse. Specialized terminology or foreign expressions were recorded with their original spelling to prevent misinterpretation. While the analysis was conducted entirely in Persian, selected representative quotes were translated into English by a professional translator specialized in nursing research for the purpose of this manuscript, ensuring the conceptual essence of the participants’ experiences remained intact.

### Data analysis

Consistent with the conventional content analysis approach, the analysis was conducted inductively without imposing a predefined theoretical framework on the data. This approach was selected because the existing literature provides limited qualitative understanding of how nurses conceptualize and enact innovative behavior in clinical practice. Accordingly, categories and conceptual relationships were allowed to emerge directly from participants’ experiences and narratives.

**Fig. 1:**
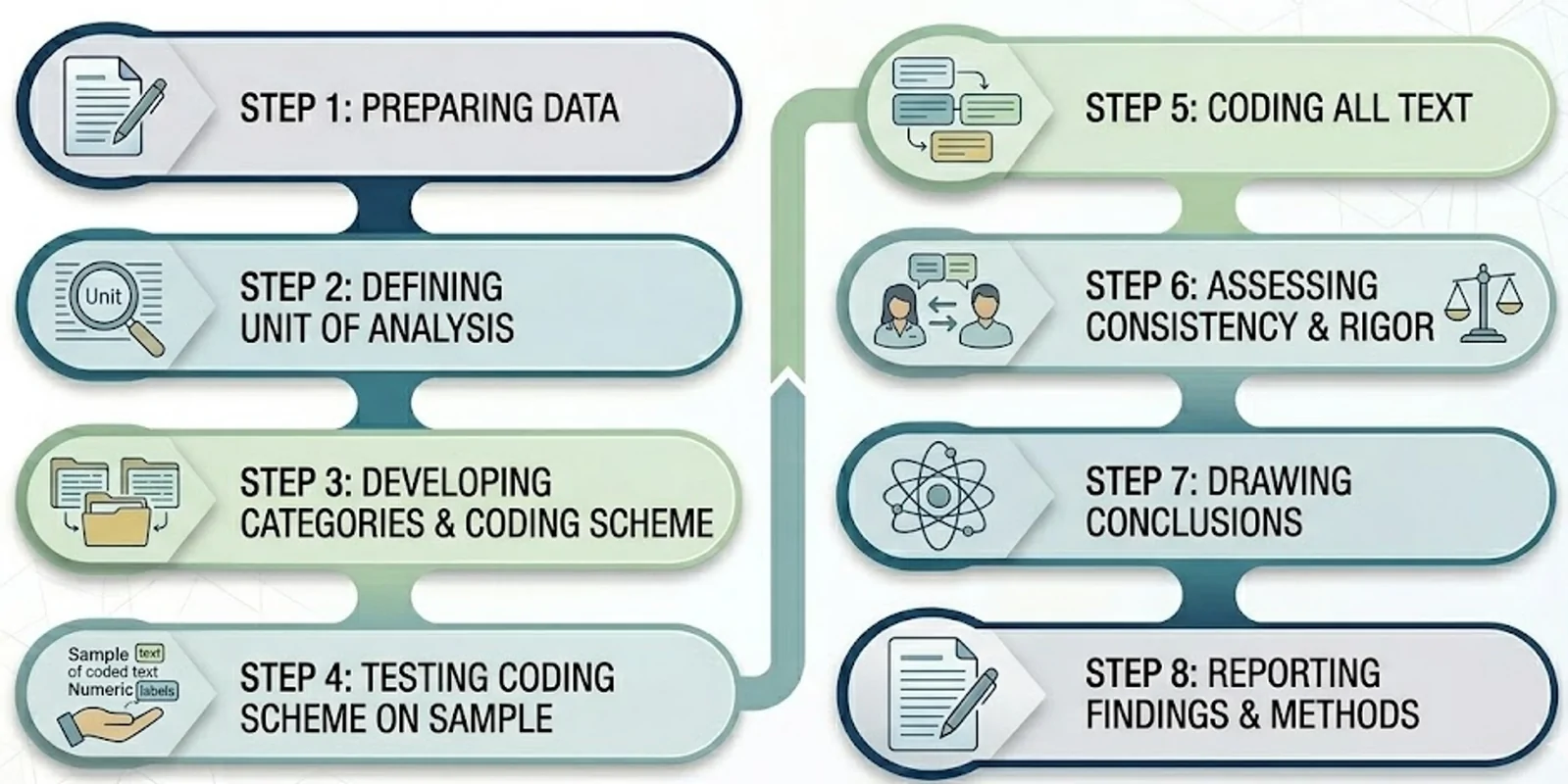
The eight-step framework for qualitative content analysis. Adapted from Zhang and Wildemuth (2016, Chapter 18). This figure illustrates the analytical trajectory from initial data preparation to the development of the final conceptual framework

Data analysis was conducted concurrently with data collection using the conventional content analysis approach described by Zhang and Wildemuth (2016) [24]. The eight-step analytical process, as illustrated in Fig. 1, was systematically followed to ensure methodological rigor. Immediately after each interview, audio recordings were transcribed verbatim in Persian and reviewed several times alongside field notes to achieve immersion in the data and develop an in-depth understanding of participants’ experiences.

The analytical process began with repeated reading of the transcripts to identify meaning units related to nurses’ innovative behavior. Meaning units consisted of words, phrases, sentences, or paragraphs that conveyed a distinct idea or experience relevant to the phenomenon under study. These meaning units were then condensed while preserving their core meaning and assigned initial codes.

Coding was performed inductively and remained close to the participants’ original expressions during the early stages of analysis. As coding progressed, codes with conceptual similarity were continuously compared, grouped, and refined to form preliminary subcategories. Through iterative discussions among the research team, related subcategories were subsequently abstracted into broader categories representing higher-level dimensions of nurses’ innovative behavior.

Data collection and analysis proceeded simultaneously, enabling emerging insights from earlier interviews to inform subsequent interviews and probing questions. Constant comparison was applied throughout the analysis to identify similarities, differences, and conceptual relationships across participants’ accounts.

To enhance analytical rigor, two members of the research team independently coded several transcripts and subsequently compared and discussed their interpretations to achieve consensus regarding coding structure and category development. Discrepancies were resolved through discussion and re-examination of the original transcripts. The full analytical process was regularly reviewed and discussed among all authors to ensure conceptual coherence and consistency with the data. MAXQDA version 22 software was used to facilitate data organization, retrieval, and management throughout the analysis process. A detailed breakdown of the analytical stages and corresponding actions is provided in Table 1.

**Table 1:** Stages of the conventional content analysis process

Analytical Stage	Description of Analytical Activities
Data immersion	Verbatim transcription of interviews, repeated reading of transcripts, and review of field notes to gain a holistic understanding of the data
Identification of meaning units	Extraction of words, phrases, sentences, or paragraphs related to nurses’ innovative behavior
Initial coding	Condensation and labeling of meaning units with inductive codes grounded in participants’ experiences
Constant comparison	Continuous comparison of codes across interviews to identify similarities, differences, and conceptual patterns
Development of subcategories	Grouping conceptually related codes into preliminary subcategories
Abstraction into categories	Integration and refinement of subcategories into broader conceptual categories representing dimensions of nurses’ innovative behavior
Team-based analytical review	Independent coding by two researchers, comparison of interpretations, discussion of discrepancies, and consensus-building among authors
Final conceptualization	Development and refinement of the final conceptual framework grounded in the data

### Accuracy and rigor

This study placed a high priority on data accuracy, adhering to Goba and Lincoln’s (1986) criteria of dependability, credibility, confirmability, and transferability [25]. Credibility was fortified through sustained immersion and engagement. Specifically, a member checking process was conducted after the initial analysis phase, where a subset of participants (*n* = 5) reviewed the preliminary findings to verify that the extracted categories and subcategories accurately represented their professional experiences.

To demonstrate reflexivity, the primary researcher maintained a reflexive journal throughout the study. This journal documented personal assumptions, beliefs regarding nursing innovation, and insights into how the researcher-participant interaction might have influenced data collection. By systematically reviewing these reflections, the research team identified and mitigated potential biases, ensuring that the conceptualization emerged directly from the participants’ perspectives. Confirmability was further ensured by two research team members who independently conducted data analysis and reconciled findings, and by an expert panel that audited the entire analytical trajectory. As depicted in Fig. 1, our analytical process was iterative and rigorous, ensuring that each step, from coding to abstraction, was systematically validated.

### Ethics approval and consent to participate

This research originates from a doctoral dissertation and received ethical approval from the institutional ethics committee of Semnan University of Medical Sciences, Iran (Ethical code: IR.SEMUMS.1401.226). All procedures performed in this study adhered to the applicable guidelines and regulations of the Helsinki Declaration. Prior to data collection, all participants were provided with comprehensive information regarding the study’s objectives and the voluntary nature of their participation. They were informed of their right to withdraw from the study at any stage without any prejudice or negative consequences. Written informed consent was obtained from all participants before the interviews were conducted. Furthermore, specific permission was secured for audio recording, and participants were explicitly informed that they could request to stop the recording at any time during the interview. To ensure anonymity and confidentiality, all personal identifiers were removed from the data during the transcription and analysis phases.

## Results

This study included 14 clinical nurses with varying levels of work experience ranging from 3 to 23 years. To address certain ambiguities and uncover deeper insights, two participants were interviewed twice. A total of 16 interviews were subsequently conducted. Further details can be found in Table 2.

**Table 2:** Characteristics of participants and interviews

Gender	Female = 5
	Male = 9
Age (years) mean (SD)	34.5 (6.008)
Marital Status	Single = 6
	Married = 8
Education	Bachelor of Nursing = 10
	Master of Nursing = 4
Work Experience (years) mean (SD)	10.785 (6.337)
Interview Duration (minutes) mean (SD)	71.187 (11.018)
Workplace Setting	Mixed (9 academic teaching hospital, 5 private)
Current Role	Mixed (8 staff nurse, 3 shift supervisor, 3 head nurse/clinical supervisor)

The encounters and viewpoints of the participants concerning nurses’ innovative behavior were methodically classified into 13 subcategories distributed across five primary domains: (1) nurses’ competencies, (2) generating care ideas, (3) feasibility of care ideas, (4) clinical implementation, and (5) promoting innovation (refer to Table 3).

**Table 3:** Main categories and subcategories

Main categories	Sub Categories
Nurses’ competencies	Nurse personality traits
	Nurse communication skills
	Nurse personal development
Generating care ideas	Problem identifying in a clinical setting
	Adapting existing solutions
	Providing novel care solutions
Feasibility of care ideas	Idea alignment with clinical principles
	Financial support
	ideas justification
Clinical implementation	Collaboration
	Initial limited implementation
Promoting innovation	Support, feedback, and continuous improvement
	Innovation sharing

### Nurses’ competencies

Within the subcategory of nurses’ competencies, the participants underscored the significance of traits such as curiosity, adaptability, willingness to take risks, a sense of responsibility, and a genuine interest in nursing. They elucidated how certain personality traits among clinical nurses contribute to the prevalence of innovative behaviors within the clinical setting. A clinical nurse articulated this sentiment, stating, “*I’m like a detective diving into patients’ problems*,* always curious to unravel what’s going on underneath. It is like unlocking doors to fresh solutions*,* so*,* it is like this journey that gets the innovation fire going in nursing*,* you know?* " (P1).

The participants highlighted the necessity of possessing nurse communication skills in nursing competencies. They discussed their ability to communicate effectively with patients and colleagues, conflict resolution skills, and persuasion. A nurse stated, *“Communicating to patients for me is like tuning into different radio stations. You gotta find the right frequency to connect. It’s not just about words; it’s about understanding. You pick up on their vibes*,* adjust your tempo*,* and suddenly*,* you’re in sync. That’s where innovation kicks in when you’re on the same wavelength.”* (P5).

The subcategory of nurses’ personal development highlighted the impact of nurses’ competencies on their innovative behaviors. Clinical nurses articulated instances of skill updating, acquisition of non-nursing skills, engagement in diverse scientific disciplines, fostering mental preparedness, and pursuing self-improvement as codes indicative of their experiences and perspectives. One of the participants said, *“To be on top of her game at work*,* a clinical nurse needs to level up personally – you know*,* self-improvement stuff. Then*,* you can bloom and bring some new ideas to the workplace.”* (P3).

### Generating care ideas

Generating care ideas is a significant part of nurses’ innovative behaviors. The participants recognized the significance of pinpointing challenges and issues within the workplace as a prerequisite for generating novel ideas. As one nurse remarked, *“As a practical nurse*,* delivering a creative solution requires me to identify the issues in patient care and locate the gaps within our department.”* (P10).

The ability to adapt existing solutions was tied to nurses generating new ideas and approaches. Nurses’ innovative behavior includes the process of borrowing and adapting existing solutions from other nursing settings. One nurse explained, *“I got buddies in the American healthcare system. Sometimes*,* I reach out to them*,* like*,* “How’d this thing go down? What’s the deal with such issues?” Their answers give me ideas for my ward.”* (P8).

Providing novel care solutions has emerged as a subcategory that drives care idea, generation. Nurses said that innovative problem solving often requires clear and collaborative innovative ideas grounded in patient needs, the introduction of novel concepts considering limitations, and the derivation of solutions from emerging issues to contribute to the development of novel care solutions. One nurse articulated, *“Whenever a new problem pops up*,* I believe there’s gotta be a fresh solution in its heart. A resourceful nurse is the one who can dig deep and find that novel fix.”* (P13).

### Feasibility of care ideas

Clinical nurses are actively assessing the feasibility of their care ideas as they navigate the path of innovative behaviors. It is imperative to assess the practicality of an innovative idea, before proceeding with its implementation. Ideas must align with clinical principles. According to one nurse, *“An innovative clinical nurse must make sure that his/her ideas align smoothly with clinical principles. It’s like ensuring all the pieces fit together logically to provide the best novel care for the clients.” (P6)*.

Participants described the essential role of financial support in rendering new ideas feasible and motivating nurses to actively participate in innovative behaviors. As one nurse put it, *“Getting finance for our new ideas is like having the right fuel for a road trip. You can have this amazing destination in mind*,* but without the gas money*,* you’re stuck in the driveway. It is the funds that open up possibilities*,* making our ideas viable*,* helping us hit the road*,* and uncover valuable solutions in patient care.”* (P7).

Clinical nurses expressed the conviction that assessing the feasibility of their innovative ideas requires comprehensive justification. This involves responsiveness to uncertainties associated with the new idea, substantiating the clinical consequences of the proposed idea, and conducting a thorough cost–benefit analysis. Overall, a holistic justification approach is deemed essential. A nurse highlighted, *“When I pitch my ideas*,* I make sure to talk about the spiritual aspects and the quality of the work that will be done at the patient’s bedside. I try to justify that my idea*,* not only helps the patient but also makes sense economically for the whole system. That’s my trick to upping the odds of getting my idea put into action.”* (P1).

Another participant emphasized the importance of evidence-based justification: *“Before I suggest any new idea to my head nurse*,* I gather whatever evidence I can*,* maybe a similar experience from another ward*,* or a small change I tested myself. If you can’t show that the idea works or at least won’t harm the patient*,* nobody will listen to you. Justification is not just about money; it’s about safety and logic too.*” (P9).

### Clinical implementation

The imperative for the collaboration of nursing leaders, physicians, and other colleagues in the implementation of innovative ideas stood out as one of the crucial topics deliberated by the participants. One nurse stated, *“Making sure your boss is on board with your fresh ideas is a big deal. I knew that if I could get her nod of approval*,* we could roll out this innovative idea*,* to improve patient care. Therefore*,* I went up and asked her to team up with me*,* and she was totally on board with it.”* (P4).

Clinical nurses involved in the study conveyed that innovative care practices are initially implemented on a trial basis with minimal scale. A nurse shared insights about her innovation: *“Okay*,* you might find it funny*,* but my first contraption was like a DIY craft. I put it together with a basic test tube. I handed it over to my colleagues for a trial run”* (P4).

### Promoting innovation

Promoting innovation is a part of nurses’ innovative behaviors. Participants emphasized innovation support, feedback and continuous improvement of the innovative idea. One of the nurses noted, *“By implementing the idea*,* multiple times*,* you can address issues based on patient feedback and its outcomes. This process contributes to the maturation of our work.”* (P7).

Nurses noted that promoting innovation serves as a powerful tool for fostering innovative behavior, enabling them to inform their colleagues about novel ideas. One nurse stated, *“Joining care innovation events and festivals can be a cool way to share fresh and effective ideas with fellow nurses. It is not just about sharing but also soaking in the creativity and buzzing with inspiration.”* (P5).

## Discussion

The present study qualitatively elucidates clinical nurses’ innovative behavior by identifying five interrelated dimensions: nurses’ competencies, generation of care ideas, feasibility of care ideas, clinical implementation, and promotion of innovation. The findings conceptualize innovative behavior as a dynamic, process-oriented phenomenon grounded in nurses’ everyday clinical practice, rather than a unidimensional or outcome-focused construct.

The first dimension, nurses’ competencies, underscores the foundational role of individual attributes and skills in enabling innovative behavior. Participants emphasized personality traits such as curiosity, adaptability, responsibility, and risk tolerance, alongside communication and continuous professional development. These findings are consistent with prior evidence demonstrating that individual personality traits and interpersonal competencies are critical antecedents of innovative behavior [26, 27]. More broadly, the literature confirms that professional competencies are essential for sustaining innovative nursing practices in complex clinical environments [28]. A key contribution of this study is the finding that competencies do not function merely as background characteristics, but as active enablers that shape nurses’ capacity to recognize problems, engage with uncertainty, and initiate innovation within clinical constraints. This finding challenges the prevailing view in nursing innovation literature that primarily emphasizes external factors such as leadership or organizational culture [5, 29]. Instead, it suggests that interventions aimed at fostering innovation should also target individual-level capacity building, including critical thinking, adaptability, and risk tolerance. Generation of care ideas emerged as a distinct and central dimension of nurses’ innovative behavior. Participants described idea generation as a deliberate and reflective process rooted in problem identification, adaptation of existing solutions, and the creation of novel care approaches responsive to patient and contextual needs. This aligns with prior research emphasizing idea generation as a core component of innovative work behavior and an essential mechanism for addressing healthcare challenges [30]. A further key contribution is the demonstration that idea generation in nursing is closely intertwined with clinical judgment, contextual awareness, and patient-centered reasoning, rather than abstract creativity alone. This contrasts with generic models of innovation that often prioritize divergent thinking or brainstorming techniques [31, 32]. In clinical nursing practice, idea generation is grounded in real-time problem-solving at the bedside, meaning that innovation cannot be separated from clinical expertise and experiential knowledge.

The assessment of feasibility of care ideas represents a critical evaluative phase that distinguishes viable innovation from impractical creativity. In this study, feasibility was conceptualized through alignment with clinical principles, availability of financial and organizational support, and the ability to justify innovations in terms of clinical effectiveness and cost–benefit considerations. This dimension reflects existing evidence that early assessment of feasibility is essential for translating innovative ideas into practice and for minimizing implementation failure [33, 34]. Participants’ emphasis on justification highlights nurses’ active engagement with both clinical and organizational logics, reinforcing the view that innovative behavior in nursing is inherently situated at the intersection of care delivery and system-level considerations. Notably, the subcategory of ideas justification, which includes clinical safety reasoning, evidence-based argumentation, and economic cost–benefit analysis, has received limited attention in prior quantitative studies on nurses’ innovative behavior. Our findings suggest that nurses actively engage in this justificatory work before proposing innovations, particularly in resource-constrained settings. This dimension may represent an underexplored but critical aspect of innovative behavior that bridges individual creativity and organizational feasibility.

Clinical implementation was identified as a transformative dimension in which innovative ideas are operationalized and tested in practice. Consistent with the distinction between creativity and innovation, implementation emerged as the defining stage through which ideas acquire tangible impact [35]. Participants highlighted collaboration with nursing leaders, physicians, and colleagues, as well as the importance of initial small-scale or pilot implementation. These findings align with existing literature underscoring teamwork and interprofessional collaboration as key determinants of successful nursing innovation [36], and with recommendations that novel clinical interventions undergo preliminary testing before broader adoption [37, 38]. This dimension underscores that nurses’ innovative behavior extends beyond ideation to include strategic engagement with organizational structures and clinical workflows.

Finally, promotion of innovation was identified as a distinct yet interconnected dimension encompassing support for innovative ideas, feedback, continuous refinement, and dissemination among peers. Participants viewed promotion not only as a mechanism for sustaining individual innovations, but also as a means of fostering a broader culture of innovation within nursing teams. This finding is consistent with evidence that supportive organizational climates and peer sharing enhance innovative work behavior among nurses [39]. Moreover, engaging in promotion and reflection appears to reinforce nurses’ creative thinking and professional growth [40, 41], suggesting that innovation is both an individual and collective process that evolves over time. While the existing literature acknowledges nurses’ potential for innovation, our study specifically identifies the conditions under which this behavior emerges. We found that the main driver for innovation was not merely organizational support, but the intrinsic clinical challenge faced at the bedside, shifting the focus from organizational climate to professional necessity as the primary incubator for innovation. Furthermore, previous studies often conceptualize nursing innovation as a formal, structured process. Our study is important because it demonstrates that for the clinical nurse, innovation is an informal, often invisible, daily practice. By categorizing these behaviors into five interrelated dimensions, we provide a framework that makes this invisible work visible to hospital management. A significant contribution of this study is the contextualization of innovative behavior within the Iranian healthcare system, a setting that faces unique resource constraints. Our findings demonstrate how nurses navigate these specific barriers, offering a context-specific conceptualization that remains relatively underexplored in the global nursing literature.

Taken together, these findings contribute to the literature by offering a comprehensive, nurse-centered conceptual framework that captures innovative behavior as a dynamic, multidimensional, and practice-embedded process. By articulating clearly defined dimensions grounded in nurses’ lived experiences, this study provides the conceptual clarity necessary for theory refinement and supports the development of context-sensitive measurement instruments for assessing nurses’ innovative behavior.

Several limitations should be considered when interpreting the findings of this study. First, the study focused primarily on individual-level experiences of nurses and did not explicitly examine external or organizational factors such as leadership styles, organizational culture, or institutional policies, which may also influence innovative behavior. Future research could integrate these contextual factors to provide a more comprehensive understanding of nurses’ innovation within healthcare systems. Second, the study was conducted within a specific cultural and healthcare context, which may limit the transferability of the findings to other settings. Finally, as the interviews were conducted in Farsi and later translated into English, some nuanced meanings or contextual expressions may have been altered despite careful translation procedures. These limitations notwithstanding, the study offers valuable conceptual insights that can inform future qualitative and quantitative investigations.

## Conclusion

This qualitative study revealed that nurses’ innovative behavior is a dynamic, five-dimensional process (competencies, idea generation, feasibility assessment, clinical implementation, and promotion). Our findings show that innovation is primarily driven by professional necessity, the everyday clinical challenges nurses face at the bedside, and that it often manifests as informal and invisible daily practice. By rendering this invisible work visible, our framework provides a grounded foundation for refining measurement tools and fostering innovation cultures, particularly in resource-constrained healthcare settings that have received limited qualitative attention to date.

## Supplementary Information

Below is the link to the electronic supplementary material.

Supplementary Material 1

## Data Availability

The datasets employed and/or scrutinized in the present study can be obtained from the corresponding author upon reasonable request. Please direct all data-related inquiries to aaghods@yahoo.com.
