# Supplementary material for: Conceptualizing nurses’ innovative behavior: a qualitative content analysis of process and dimensions
Source: BMC Health Serv Res. 2026 Aug 14;26:1584. doi: 10.1186/s12913-026-15320-5 (PMC13760860; doi:10.1186/s12913-026-15320-5)
Supplement: Supplementary file 1 [file 12913_2026_15320_MOESM1_ESM.docx]

**Semi-Structured Interview Guide Used to Explore Nurses’ Innovative Behavior in Clinical Practice**

**Step 1: Introduction Script (Read to Participants)**

Thank you for agreeing to participate in this study. The purpose of this interview is to explore your experiences and perspectives regarding innovative behavior in clinical nursing practice. There are no right or wrong answers. We are interested in your personal experiences and insights. With your permission, this interview will be audio-recorded to ensure accuracy. All information will remain confidential and anonymized.

**Step 2: Understanding the Participant's Background**

1. Can you briefly describe your current role as a clinical nurse in a healthcare organization, including your areas of specialization and responsibilities?

2. How long have you been working as a clinical nurse, and what is your educational background in nursing?

**Step 3: main topics**

Exploring Nurses' Innovative Behavior

- How do you define "innovative behavior" in nursing, based on your experiences?
- Can you describe your experiences with innovation in your clinical nursing practice?
- In your opinion, how important is innovative behavior for improving patient care and outcomes?
- Can you share any instances where innovative behavior directly contributed to improved patient outcomes or care quality?

**Follow-Up Questions (Used Flexibly Based on Participants’ Responses)**

Can you provide an example of a situation in which you did something new or differently in patient care?

What led you to take that action?

How did you experience that situation?

What happened afterward?

How did others respond?

What factors influenced your ability to act in that way?

How did you feel about the outcome?

Have you had similar experiences in other situations?

**Probing Prompts (as Needed)**

Can you explain that further?

Could you describe that in more detail?

What do you mean by that?

Can you give another example?

What was challenging in that situation?

What helped you in that process?

**Step 4: Conclusion and Closing**

- What are your overall thoughts on the findings discussed in this interview, and how do you believe they relate to the broader healthcare landscape?
- Is there anything else you would like to add or any insights you think are important for this study?

**Step 5: Thank You and Debriefing**

- Thank the participant for their time and willingness to participate.
- Reiterate the confidentiality of the interview data and how it will be handled.
- Provide contact information for any follow-up questions or concerns.
- Confirm that the participant has no further questions or comments.

**Interviewer Notes**

The interview should follow a flexible and inductive approach.

Questions should be adapted based on participants’ narratives.

No predefined dimensions or conceptual framework should impose during the interviews.

The focus should be on eliciting rich descriptions of lived experiences.
